# Community Health: A Critical Approach to Addressing Chronic Diseases

**Published:** 2007-09-15

**Authors:** Rachel Davis, Lissette M. Flores, Patti Culross

**Affiliations:** Prevention Institute; Prevention Institute, Oakland, California; Prevention Institute, Oakland, California

## Abstract

Many public health solutions to chronic diseases involve individual lifestyle choices: eating more healthfully, increasing physical activity, and quitting smoking. This approach neglects barriers in the community environment that make modifying unhealthy behaviors challenging. Addressing environmental barriers is an essential strategy to supporting behavioral changes. Changing community environments that contribute to unhealthy behaviors can improve community health.

Community indicator reports can be used to strengthen community environments for optimum health. The reports are comprehensive evaluations of community well-being that reflect community factors that influence health. Prevention Institute studied community indicator reports for The California Endowment and produced *Good Health Counts: A 21st Century Approach to Health and Community for California. *This commentary on that document highlights recommendations for the use of community indicator reports.

## Background

Thinking about health as a function of environmental influences in our communities shifts the focus from individual behaviors to health needs and barriers that can be addressed through broad strategies such as policy change. Good health is a cornerstone of community members' quality of life and productivity and of the community's economy. Community indicator reports and report cards can be used to enhance the process of restoring good health in a community.

Most people understand prevention of chronic disease on an individual level. Accordingly, many public health solutions involve individual and lifestyle changes: eating more healthfully, increasing physical activity, and reducing or quitting smoking. Environmental barriers in the community can make modifying unhealthy behaviors challenging. Poor environmental quality; inadequate access to affordable, nutritious food; and safety issues often make healthy living impractical, particularly in low-income communities and communities of color. Although education can play a role in influencing individual behavioral choices, addressing environmental variables is an essential strategy to supporting behavioral change. Analyzing the underlying causes of inadequate diets and low levels of physical activity, for example, shows that community conditions play an important role in shaping health-related behaviors. To have an impact on rates of chronic disease, community environments must support and encourage healthy habits.

The Institute of Medicine affirmed the need to focus on changing the environment in order to foster behavior change, asserting:

To prevent disease, we increasingly ask people to do things that they have not done previously, to stop doing things they have been doing for years, and to do more of some things and less of other things. . . . It is unreasonable to expect that people will change their behavior easily when so many forces in the social, cultural, and physical environment conspire against such change (1).

For example, environments supportive of reducing asthma, lung cancer, and cardiovascular disease would address the availability of tobacco, safe and affordable access to healthy food, safe access to physical activity, social cohesion and environmental design that encourage physical activity, and social and behavioral norms that encourage healthy habits and discourage unhealthy ones (Table). Understanding risk behaviors associated with chronic diseases and the elements of the community environment that contribute to those risk behaviors enables communities to develop strategies for addressing environmental factors. Indicators also serve as a tool for tracking progress in altering the community environment to support better health outcomes.

By paying attention to community environments that contribute to unhealthy behaviors, community indicator reports are an important tool for improving community health. Community indicator reports are comprehensive evaluations of community well-being that include multiple categories, reflecting various aspects of the ways people live and the living conditions in neighborhoods, cities, counties, states, or countries. They can be used to broaden the definition of health, develop local priorities, track progress, and bring disparate groups together in support of community health (2). In 2006, Prevention Institute studied community indicator reports for The California Endowment and produced *Good Health Counts: A 21st Century Approach to Health and Community for California *(3). This commentary on that document highlights its recommendations for the use of community indicator reports.

## Methods

Prevention Institute reviewed 79 community indicator reports and 9 popular-culture report cards and interviewed 64 key informants. A review of literature also informed the study. As defined in *Good Health Counts*, the term *community indicator reports* means published reports that use a carefully selected set of indicators to track the social, health, and economic conditions in a defined geographic area. *Report cards* are community indicator reports that use letter grades or rankings for each report element.

Community indicator reports use different naming conventions to categorize information. For example, the term *indicator* can mean the actual data or can refer to an aggregation of data measures. No standard definition was found among reports. In this paper, an* indicator* is defined as a construct consisting of more than one measure, with *measures* being the actual data. Indicators either relate to the entire population or subpopulation or, in the case of a *performance measurement,* the effectiveness of a service or program.

The reports reviewed for *Good Health Counts* were self-identified as indicator reports, were available on the Internet, and were published in English. Additional reports were suggested by individuals or mentioned in other indicator reports or in the community indicators literature. The geographic scope was limited to the United States, Canada, the United Kingdom, Australia, other English-speaking countries, Europe, Japan, and Hong Kong. Reports from international nongovernmental agencies, such as the World Health Organization, were also reviewed. 

### Community indicator reports

Community indicator reports share many common features and criteria for development, even though they often differ in their approaches. The reports reviewed were categorized as quality of life, sustainability, health status, social well-being, and government performance. Many of the reports were not specifically referred to as community health reports, yet they offered valuable information about one or more elements in the community environment that contribute to the health of the community they describe.

### Report cards

Generally, community indicator reports are comprehensive summaries of community conditions, including health status, that include explanations about what each indicator means and why it is important. Occasionally, community report cards will feature highlights from larger reports and provide additional meaning to the information by adding grades, rankings, or comparisons as a way to track progress or performance over time in a way that readers recognize. They are, by definition, selective in what they report, but the use of grades and other types of judgments can be effective in improving community health outcomes. For example, providing incentives for change, especially when the grades identify key indicators and establish priorities, contributes to ownership and to action around potential solutions.

## Recommendations

### Elements of an effective report tool

Although community indicator reports and report cards serve various purposes, a number of elements emerged that facilitate their use by advocates, community members, health departments, and other stakeholders (Box 1). The most comprehensive and valuable reports are able to monitor trends over time and offer some interpretation about the magnitude and direction of any changes. Simply making indicators available will not result in change. Effective indicator reports frame the information in a way that can lead to action; they identify relevant policies and steps that can be undertaken to improve the indicator. Reports and report cards also work best in a context of accountability (i.e., when the agencies or organizations responsible for acting on the information are clearly identified).

**Box 1. Elements of an Effective Report Tool**

- Tracks progress and trends
- Is actionable
- Establishes accountability
- Focuses on community assets
- Captures what is important
- Is grounded in a plausible theory of change
- Uses credible and trustworthy data
- Uses meaningful language
- Is accessible and user-friendly
- Is values-based

### Elements of an effective process

Community indicator reports facilitate community improvement in a number of different ways. They may foster community engagement and collaboration, improve health care quality, identify agendas for public resource distribution, set baselines for government performance, monitor progress in government performance or community health and well-being, inform public policy development and advocate for specific policies, or do a combination of these. Some reports focus on improving community health through a particular sector, whereas others suggest multisector collaborations to achieve the desired outcome. 

**Box 2. Elements of an Effective Process**

- Presents a vision for community health
- Focuses goals based on key opportunities
- Fosters collaboration based on relationships between sectors
- Selects key indicators for maximum leverage in a given sector
- Establishes accountability
- Makes a commitment to data source development
- Promotes and continues to seek ongoing community input

The process of developing community indicators is aimed at report creation, but several additional elements of the process are important for a successful outcome (Box 2). One of the most important considerations is a commitment to ongoing community input. Community input ensures that reports and the process of developing reports reflect local priorities and keep the meaning of indicators transparent and clearly understood by populations for whom the report is intended. A related process involves the inclusion and participation of various sectors that can affect community health, including public health, transportation, education, housing, and employment, among others. Community members who are accountable for making progress should be included early in the process, allowing trust and relationships to develop. The process of developing a community indicator report can facilitate dialogue on issues that matter, translate collaboration into a meaningful product, and allow communities to think through a vision for a healthy future. The process of taking an interest in and contributing to the improvement of the conditions for health in a community can also be valuable to a community's overall health. The process is what makes the difference; the report is a tool that results from the process.

### Community indicator reports in action: three case studies

The following case studies illustrate many of the possible outcomes of community indicator reports. In West Oakland, California, a powerful coalition of local organizations worked together to rid the community of a source of air pollution. Their efforts educated and empowered the community, forming the foundation for further community action. In King County, Washington, the local public health department initiated a report that revealed county strengths and needs, enabling the community to identify its priorities, guide funding decisions, and suggest actions that strengthened the community environment. The example of Jacksonville, Florida, shows that long-term tracking of community indicators can influence public policy and planning in support of comprehensive social services.

#### West Oakland, California

Released in 2002, *Neighborhood Knowledge for Change: the West Oakland Environmental Indicators Project* (4) is a collaboration between the 7th St/McClymonds Corridor Neighborhood Improvement Initiative and the Pacific Institute for Studies in Development, Environment, and Security. For 2 years these two organizations, West Oakland residents, and other partners worked together to research and identify 17 indicators to monitor environmental, health, and social conditions for the West Oakland neighborhood. West Oakland residents used the report data to garner support from the media, elected Oakland officials, and the public health community to close down the Red Star Yeast factory, the largest toxic air polluter in West Oakland. The West Oakland Environmental Indicators Project is now an independent nonprofit organization and continues to work on community issues, such as land use, air quality, and the effect on the community of the movement of goods at the Port of Oakland.

#### King County, Washington

In 2005, the Seattle-King County Public Health Department published *Communities Count: Social and Health Indicators Across King County *(5). Now in its third generation, this report was developed to identify the strengths and needs in Seattle-King County. Over the years the report has influenced many community endeavors. For example, the United Way looks to the report for direction in strategic planning for safety services and programs on focus areas such as early childhood development and homelessness. In 2002, the Seattle Foundation used the report to inform local donors on where to invest in the community. One indicator, the measure of time parents spend reading to their children, motivated the public health department to facilitate a South County coalition of library staff; employees of the Special Supplemental Nutrition Program for Women, Infants, and Children (WIC); and others to promote reading to children among WIC clients. Community-based organizations use data in the report for grant applications; the county's Children and Communities Commission (6) requires that grant applicants choose an indicator from the report to address when seeking funding.

#### Jacksonville, Florida

Now in its 22nd year, the *Quality of Life Progress Report* (7), produced annually by the Jacksonville Community Council, Inc (JCCI), is one of the oldest continuous reports of its type in the country. Much of the report's influence results from JCCI's early collaboration with the Jacksonville Chamber of Commerce and a key funder, the United Way of Northeast Florida. In general, the report functions as an annual accountability measure of government and community services that underscores the value of data-driven decision making. It has become an essential tool in funding decisions by the United Way and has been used in government benchmarking initiatives by the Jacksonville Chamber of Commerce. The report informed the creation of new early childhood development and senior programs in the community and serves to monitor the impact and effectiveness of services provided by various community-based organizations, not only among their own client population but also in the larger community.

## Conclusion

Developing a strategy for promoting community health requires understanding the places where people live, work, and play. Community indicator reports can be an important tool to assess and strengthen community environments for good health, enabling people to be productive, to learn, and to live healthy lives with dignity and self-determination.

Over the past 2 decades, community indicators and indicator reports have proliferated. Three broad themes are associated with the widespread use of indicator reports: the existence of many local initiatives aimed at improving general community well-being and quality of life; the evolution of a broadened definition of health, which has shaped the content of indicators; and the influence of the expanded availability and use of data.

The quality and accessibility of data have improved markedly over the past 25 years, which has strengthened the capacity to measure and monitor health. Information technology, combined with new and expanded sources of data, has transformed health assessment dramatically.

With these increases in data capacity, community indicator reports can facilitate community improvement by fostering community engagement and collaboration, framing accountability, informing policy, and gaining media attention. As evidence mounts about elements in the community environment that contribute to the onset or prevention of chronic disease, indicators are a valuable tool to track progress and hold ourselves accountable for addressing the critical determinants of chronic disease.

## Figures and Tables

**Table.** Community Environments, Mediating Indicators, and Chronic Disease, United States, 2006

**Elements of the Community Environment**	**Sample Indicators**	**Significance and Related Chronic Diseases**
***Place *** refers to the physical environment in which people live, work, play, or go to school. Associated community factors include What is sold and how it is promoted Look, feel, and safety Parks and open space Getting around Housing Air, water, and soil Arts and culture	Residents who eat 5 servings of fruits and vegetables per day. No. and types of supermarkets Alcohol outlet density Tree planting Abandoned buildings Life on the street (e.g., foot traffic) Places to play Perceived safety Safe, clean parks Bikeable and walkable streets Public transport availability Travel time to work Average weekday bus ridership per 1,000 people Housing affordability Owner-occupied housing Density/people per unit Local wild salmon runs Air quality Beach closures Water quality Pollution in neighborhoods Participation in arts and culture	These factors and sample indicators are associated with eating and nutrition, alcohol consumption, physical activity levels, and level of exposure to toxins, all of which can increase or reduce the risk of multiple forms of chronic disease, such as asthma, cancers, cardiovascular disease, and diabetes.
***People *** refers to the relationships between people, the level of engagement, and norms, all of which influence health outcomes. Associated community factors include Social networks and trust Participation and willingness to act for the common good Acceptable behaviors and attitudes	Neighborhood involvement Local and indigenous leadership Sense of community Commitment to community among its members Trust Voter activity Volunteerism Tendency to intervene or act to achieve community aims Availability of alcohol and cigarettes to minors	These factors and sample indicators are associated with social support for, and norms related to, multiple behaviors (e.g., eating, drinking, physical activity, sexual activity) as well as the capacity to make community-level changes that can improve health outcomes (e.g., reduce toxic exposure, obtain land for a new park, open a grocery store). Related chronic diseases include cancers, cardiovascular disease, diabetes, HIV/AIDS, and mental health problems.
***Equitable opportunity *** refers to the quantity and equitable distribution of opportunities and resources. Associated community factors include Racial justice Jobs and local ownership Education	Racially balanced schools Perceptions of racism Business ownership Percentage at or above living wage Unemployment and employment rates Local ownership of assets Reading level School success (dropout/graduation) Percentage of parents reading daily to their children	Access and equity affect health in fundamental ways over a lifetime, such as through stressors associated with poverty and racism. These factors are correlated with multiple chronic diseases.
